# Acceptability and feasibility of a screening protocol for antenatal depression (SPADe) in Blantyre District, Malawi

**DOI:** 10.1186/s12888-022-04195-5

**Published:** 2022-08-12

**Authors:** Genesis Chorwe-Sungani, Modesta Mwagomba, Ellen Chirwa, Diana Jere, Jennifer Chipps

**Affiliations:** 1 Kamuzu University of Health Sciences, School of Nursing, P/Bag 360, Blantyre, Malawi; 2 District Nursing Officer, Blantyre District Health and Social Office, Chipatala Avenue, Blantyre, Malawi; 3 Kamuzu University of Health Sciences, School of Maternal Neonatal and Reproductive Health, P/Bag 360, Blantyre, Malawi; 4 University of the Western Cape, School of Nursing, Private Bag X17, Bellville, 7535 South Africa

**Keywords:** Acceptability, Antenatal depression, Feasibility, Midwives, Screening, SPADe

## Abstract

**Background:**

Depression is one of the most common perinatal mental health problems that affect pregnant women. Antenatal depression can adversely affect the well-being of the pregnant woman and her foetus. Depression is rarely detected by midwives due to the unavailability of relevant screening instruments in Malawi. A Screening Protocol for Antenatal Depression (SPADe) was developed and recommended for possible use to screen for depression in antenatal clinics in the country. The acceptability and feasibility of using the SPADe protocol to screen for depression has not been established. The aim of this study was to assess the acceptability and feasibility of screening for depression by midwives using SPADe in antenatal clinics in Blantyre district.

**Methods:**

This study used a quantitative survey design to collect data among 60 midwives in three antenatal clinics in primary care settings. All inclusive sampling of all 60 midwives were used. The Structured Assessment of FEasibility and Ottawa Acceptability of Decision Rules Instruments were used to collect the data. Descriptive statistics and Chi square tests were used to analyse the data.

**Results:**

This study found that it was feasible to implement SPADe and the following enablers for screening depression had the highest ratings: the SPADe is applicable to pregnant women (M = 3.9, sd = 0.4); the intended goal of the SPADe matches the prioritised goals of Malawi Ministry of Health (M = 3.9, sd = 0.5); and the SPADe is likely to be effective (M = 3.8, sd = 0.6). On the other hand, barriers for implementing the SPADe were: the need for specific training to deliver the SPADe (M = 3.7, sd = 0.7); ongoing support and supervision (M = 3.5, sd = 0.8); and additional resources (M = 3.0, sd = 0.9). This study also found that the implementation of the SPADe was acceptable to respondents. The overall mean score for respondents on acceptability of screening antenatal depression using SPADe was found to be high (M = 4.6, sd = 0.6). However the differences in the respondents’ mean scores on acceptability of screening for depression in antenatal clinics using SPADe in relation to their demographic characteristics were not significant (*p* > .05).

**Conclusion:**

This study suggests that midwives feel that it is feasible and acceptable for them to implement the SPADe in antenatal clinics with ongoing training, support and clinical supervision.

## Background

Midwives are expected to screen all pregnant women for depression [1, 2] to improve detection of antenatal depression [3]. Antenatal depression is a type of depression which occurs during pregnancy [4]. In Malawi, the prevalence of antenatal depression (25.8%) [5] is lower than that recorded in South Africa (35%) [6]. The condition can lead to a lower uptake of antenatal services [7], as well as adverse effects on the health of the pregnant woman and the foetus [8–11]. Pregnant women should be screened for depression using a standard and valid instrument [12]. Screening for depression during pregnancy may reduce depressive symptoms among these women [13]. However some midwives may consider screening for antenatal depression to be too demanding and requiring too much effort[14] suggesting limited feasibility and acceptability of depression screening in antenatal clinics. Conversely, evidence from the United Kingdom [15] and Sri Lanka [16] showed that midwives considered screening for depression in antenatal clinics as feasible and acceptable. In this study, feasibility refers to a process of determining whether an intervention can be shaped to be relevant and sustainable by identifying what aspects need modification and how changes might occur [17]. Acceptability refers to what extent a new idea, programme, process or measure is judged as suitable, satisfying, or attractive to programme deliverers or programme recipients [17].

In low-resource countries, comprehensive antenatal services focusing on the identification and treatment of antenatal depression are desirable since prenatal care is often the first and only time many women encounter the health care system [18]. For this reason, it is preferable to start implementing maternal mental health screening and treatment as part of prenatal appointments [19]. Despite this, due to a lack of relevant depression screening instruments in Malawi's maternity clinics, midwives regularly miss antenatal depression [20]. In looking at the evidence of acceptability and feasibility from international studies, a systematic review (included studies = 29) reported that healthcare professionals felt screening for antenatal depression in various care settings was an acceptable process [21]. Similarly, a study using a sample of 445 pregnant women found that it was also feasible to integrate screening for antenatal depression in maternity care services in Spain [22]. In Malawi, maternal mental health is integrated in the general health care system at policy level [23], so that pregnant women have easy access to mental health services.

A previous study in Malawi created a Screening Protocol for Prenatal Depression (SPADe) and proposed it as suitable for use in antenatal clinics [24]. The name “SPADe” draws its figurative meaning from an instrument that is used to dig the ground, the ‘spade’[24]. As a screening protocol, the SPADe aims to make maternal mental health care more available to pregnant women. It is based on the idea that frequent screening in antenatal clinics increases the detection of pregnant women with depression, and that midwives can be trained to screen for antenatal depression, provide psychoeducation, and make appropriate referrals. The protocol includes an algorithm for screening and pathways to treatment for pregnant women. It is critical that midwives in local antenatal clinics receive SPADe training. This could aid midwives in incorporating depression screening into prenatal services [25] and providing organized mental health care [26]. Despite this, the acceptability and feasibility of utilising SPADe to screen for prenatal depression by midwives has not been tested. As a result, the aim of this study was to assess if screening for depression by midwives using SPADe at antenatal clinics in the Blantyre district was feasible and acceptable.

## Methods

### Study design

A survey was used to collect quantitative data to assess feasibility and acceptability of screening for depression by midwives using the SPADe in antenatal clinics in Blantyre district.

### Study setting

The study setting comprised the following health centres in rural and urban areas of Blantyre district: Chileka, Mpemba and Ndirande. Antenatal clinics in these health centres offer free health services. The clinics were run by at least one midwife and an assistant per clinic, who attend to pregnant women five days a week from morning to noon. The antenatal registers showed that attendance at these facilities ranged from 1292 to 11,498 visits between June 2018 to July 2019. These clinics were selected because they have been working with the researcher over time and were familiar with maternal mental health in some capacity and the screening instrument.

### Study population and sample

The target population for this study were all 60 midwives (Male = 5, Female = 55) working at Chileka, Mpemba and Ndirande Health centres in Blantyre district. The cadres of midwives included: Community Midwives (certificate in community midwifery), Nurse Midwife Technicians (college Diploma in Nursing and Midwifery), Registered Midwives (Bachelor of Science in Nursing and Midwifery or Bachelor of Science in Nursing plus a University Certificate in Midwifery or University Diploma in Nursing and Midwifery or University Diploma in Nursing plus a University Certificate in Midwifery) [27]. These midwives resided within the communities they served. People in these communities have an average annual income of US$100-US$200 per year with 51.5% living below the poverty line [28] (Male employment rates = 66.7%, Female = 44.1%). The overall prevalence of Human Immunodeficiency Virus (age = 15–49 years) in this area is 12.8% (Men = 9.2%, Female = 15.7%) [29]. This study used all-inclusive sampling, including the total population of midwives (*N* = 60) who were working in antenatal clinics (Chileka, *n* = 19; Mpemba, *n* = 15 and Ndirande, *n* = 26) during the study period; and who had received two days training in screening for antenatal depression using the SPADe.

### Measures

All respondents anonymously completed a questionnaire at the end of two days training on the use of SPADe. The questionnaire comprised questions regarding demographic characteristics (age, workplace, gender, cadre, qualification, marital status and work experience), questions from an adapted Structured Assessment of FEasibility (SAFE) Version 1.1 [30] and questions from the Ottawa Acceptability of Decision Rules Instrument (OADRI). The SAFE was used to assess the extent to which SPADe was feasible for implementation in antenatal clinics. SAFE was found to have an good interrater (κ = 0.84, 95% CI 0.79-0.89) and test–retest reliability (κ = 0.89, 95% CI 0.85-0.93) when it was developed [30]. Respondents were asked to indicate their level of agreement with each of the 16 statements by choosing one of the following possible responses: Yes (4), Partial (3), No (2) or Unable to rate (1). One rating summary score (mean) was calculated for each item. The highest possible summary score for each item was 4. No overall summary score of the tool was calculated because barriers and enablers differ in their importance depending on the context. In addition to answering prospective questions on feasibility, the participants also responded to the Ottawa Acceptability of Decision Rules Instrument (OADRI) that was designed to measure the acceptability of clinical decision rules [31].

The OADRI was used to assess acceptability of the SPADe among respondents who were asked to indicate their level of agreement with each of the 12 statements on a 6-point likert scale ranging from 1 (strongly disagree) to 6 (strongly agree) or indicating “no opinion/don’t know”. The first 7 items were phrased such that a higher number indicated greater acceptability while the last 5 were phrased so that a higher number indicated less acceptability. The final total score of the instrument consisted of the average of all 12 items (ranging from 0 to 6). Non-completed items were excluded from the final total scores. The average of the remaining items served as the instrument’s score. Respondents who completed less than 8 of the 12 items were considered as not having completed the instrument and were excluded from the final analyses. Items for which “No opinion/Don’t know” was selected were coded as the middle of the scale [31]. The OADRI proved to be reliable (Cronbach’s Alpha > 0.8) when it was developed [31] and this study also found it to be reliable with good internal consistency in the local setting (Cronbach’s Alpha = 0.8). The OADRI was chosen for use in this study following a literature review though no documentation reported use of the OADRI in Sub-Saharan Africa.

### Data collection

The lead author with the help of two research assistants (Registered Midwives) collected data from the respondents in August 2020. Prior to data collection, the research assistants received two days training on an overview of the research study, data collection and ethical issues related to the study. The research assistants explained the study to all respondents and invited them to participate in this study. Those who accepted to participate completed a written consent form before they participated. A self-administered structured questionnaire was handed out to each respondent with a request to be completed immediately. All questionnaires were collected by the research assistants immediately after completion. All the midwives (N = 60) from the three selected health centres were invited to a venue in Blantyre District in August 2020 fortwo days training on screening for depression using the SPADe. The training was facilitated by the researcher in two groups (30 participants each) to observe the COVID-19 restrictions for meetings in effect at that time.

### Data analysis

Data were analysed using Statistical Package for Social Sciences (SPSS) version 25. Descriptive statistics for barriers and enablers for implementation of SPADe were computed. The summary item scores for feasibility on the SAFE were computed to identify enablers and barriers for use of SPADe. Each item that had a Mean score ≥ 3 was considered as either an enabler or a barrier.

The OADRI average item scores for acceptability of SPADe were calculated and greater acceptability was indicated by higher scores (≥ 4). Chi square test was used to test if there were any significant differences in the acceptability scores in relation to demographic characteristics.

### Ethics

This study received ethics approval from College of Medicine Research and Ethics Committee with reference number: P.02/20/2935.

## Results

### Demographic characteristics of respondents

All invited midwives (*n* = 60) completed a questionnaire (100% response rate). Nearly half of the respondents were from the Ndirande Health Centre (43.3%, *n* = 26) (Table 1). Respondents’ ages ranged from 23 to 58 years (M = 33.9, sd = 8.4 years). The majority of these respondents (91.7%, *n* = 55) were female, with nearly three quarters being Nurse Midwife Technicians (71.7%, *n* = 43) and nearly two thirds reported having a Diploma (65%, *n* = 39) as the highest qualification.

**Table 1:** Demographic characteristics

	Item	Mean	sd
Age		33.9	8.4
Work experience		8.2	7.3
Health centre			
Ndirande	26(43.3)		
Chileka	19(31.7)		
Mpemba	15(25)		
Gender			
Female	55(91.7)		
Male	5(8.3)		
Cadre			
Nurse Midwife Technician	43(71.7)		
Community Midwife	8(13.3)		
Registered Nurse Midwife	7(11.7)		
Enrolled Nurse Midwife	2(3.3)		
Highest qualification			
Diploma	39(65)		
Certificate	12(20)		
Degree	9(15)		
Marital status			
Married	37(61.7)		
Not married	23(38.3)		

### Feasibility of screening for depression using SPADe

This study revealed that respondents identified a variety of enablers (Table 2) and barriers (Table 3) for the implementation of a protocol for the screening of depression in antenatal clinics. The following enablers had the highest ratings by respondents: the SPADe will be applicable to pregnant women (M = 3.9, sd = 0.4); the intended goal of the SPADe matches with the prioritised goals of Ministry of Health (M = 3.9, sd = 0.5); and the SPADe is likely to be effective (M = 3.8, sd = 0.6) (Table 2). However, the nature of the SPADe being reversible was considered as a least enabler by respondents (M = 3.1, sd = 1.2).

**Table 2:** Respondents’ scores of enablers of SPADE based on SAFE

Item	Scores	
	**Mean**	**sd**
Will be applicable to population of interest (e.g. pregnant women using antenatal clinics)	3.9	0.4*
The intended goals of the SPADe match the prioritised goals of the Ministry of Health	3.9	0.5*
Is likely to be effective (e.g. evidence based and expected to produce positive outcomes)	3.8	0.6*
Is flexible (e.g. can it be tailored to the context and situation)	3.7	0.6*
Can be piloted	3.5	1,0*
Is manualised	3.5	1.0*
Will be cost saving	3.2	1.0*
Is reversible	3.1	1.2*

^*^ = Enabler at cutoff ≥ 3

**Table 3:** Respondents’ scores of barriers of SPADe based on SAFE

Item	Scores	
	**Mean**	**sd**
Staff will require specific training to deliver the?? SPADe	3.7	0.7*
Will require ongoing support and supervision	3.5	0.8*
Will require additional material resources	3.0	0.9*
Will require additional human resources	2.8	1.0
Is complex	2.6	0.9
Will be time consuming to implement	2.8	0.8
Will be costly	2.3	0.7
There are known serious or adverse events associated with the SPADe	2.2	0.7

^*^ = Barrier at cutoff ≥ 3

This study found three barriers for screening for depression in antenatal care settings using SPADe (Table 3) namely: “staff will require specific training to deliver the SPADe (M = 3.7, sd = 0.7)”; “the SPADe will require ongoing support and supervision (M = 3.5, sd = 0.8)”; and “the SPADe will require additional material resources (M = 3.0, sd = 0.9)” (Table 3).

### Acceptability of screening for depression using SPADe

The overall mean rating on acceptability of screening antenatal depression using SPADe was high (M = 4.6, sd = 0.6) but varied ranging from M = 3.2, sd = 1.3 to M = 5.4, sd = 0.8 out of a possible mean score of 6 (Table 4). However, the ratings of the following items were higher than overall mean score: “SPADe will be useful in my practice” (M = 5.4, sd = 0.8); “Clients will benefit from use of SPADe” (M = 5.3, sd = 1); “SPADe will be easy to use” (M = 5, sd = 1); “Wording of SPADe is clear and unambiguous” (M = 5, sd = 1); “SPADe will be easy to remember” (M = 4.9, sd = 1.1); “Environment I work in will not make it difficult to use SPADe” (M = 4.8, sd = 1.4); and “My colleagues will support use of SPADe” (M = 4.7, sd = 1).

**Table 4:** Respondents’ mean scores of acceptability on OADRI

Item	Scores	
	**Mean**	**sd**
SPADe will be useful in my practice	5.4	0.8
Clients will benefit from use of SPADe	5.3	1
Wording of SPADe is clear and unambiguous	5.1	1
SPADe will be easy to use	5	1
SPADe will be easy to remember	4.9	1.1
Environment I work in will not make it difficult to use SPADe	4.8	1.4
My colleagues will support use of SPADe	4.7	1
SPADe will result in improved use of resources	4.6	1.3
SPADe will account for important clinical cue??	4.4	1.4
I am already using another protocol or similar strategy	4.2	1.6
Evidence supporting SPADe is not flawed	3.6	1.2
SPADe will not increase the chance of lawsuits	3.2	1.3
Overall summary Score	4.6	0.6

There were no significant differences in respondents’ mean scores on acceptability of screening for depression in antenatal clinics using SPADe in relation to their demographic characteristics (*p* > 0.05) (Table 5).

**Table 5:** Respondents mean scores on acceptability of screening for depression inrelation to their demographic characteristics

Demographic characteristics	Scores		Statistic
	Mean	sd	$\chi$^2^, p
Health Centre			0.2, .922
Mpemba	4.7	0.7	
Ndirande	4.7	0.7	
Chileka	4.4	0.5	
Age			0.4, .554
≤ 32 years	4.6	0.5	
≥ 33 years	4.5	0.8	
Gender			0.3, .592
Male	4.9	0.6	
Female	4.6	0.6	
Cadre			1.2, .741
Nurse Midwife Technician	4.7	0.7	
Registered Nurse Midwife	4.7	0.3	
Enrolled Nurse Midwife	4.4	0.9	
Community Midwife	4.3	0.3	
Highest qualification			0.8, .860
Certicicate	4.3	0.6	
Diploma	4.6	0.6	
Degree	4.9	0.6	
Marital status			1.9, .161
Married	4.6	0.7	
Not married	4.7	0.5	
Work experience			0.4, .554
≥ 7 years	4.7	0.8	
≤ 6 years	4.5	0.4	

## Discussion

Midwives are expected to be crucial in screening mental disorders and provision of initial psychosocial care for women in Africa [32] although it is not part of routine maternity care [33] in Malawi. This study suggested that screening for depression by midwives using the SPADe in antenatal clinics was both feasible and acceptable locally. Consistent with this study, screening of mental health problems by midwives in antenatal clinics in Mali was found to be feasible and acceptable [32]. The Malawi Ministry of Health prioritised the goal of treating depression at the primary level of care [34]. However antenatal clinics in primary care in the country have inadequate staff and high workloads, making it difficult to deal with mental health issues. Midwives also lack instruments for screening depression in these settings [35, 36]. There is an urgent need for locally validated screening instruments that are brief and easy to use by various cadres of health care providers in predominantly rural African countries with limited health facilities, inadequate staff and high workloads [37]. Screening protocols for antenatal depression could help midwives to implement effective interventions systematically without adding to their workload [25] in these busy antenatal clinics. Nonetheless, it is important to assess the feasibility of implementing a screening instrument in settings where mental health workers are scarce in order to promote its uptake and usage among health care providers [38]. This study suggested that implementaion of the SPADe was feasible because it was applicable to pregnant women and its intended goal matched with the prioritised goal of the Malawi Ministry of Health to treat depression. This is consistent with evidence from Africa which showed that integrating screening, brief interventions and referral to treatment into the midwife obstetric services can facilitate access to mental health services by pregnant women [39]. As such one can anticipate that the Malawi Ministry of Health would likely allocate the resources for implementing SPADe because the screening for antenatal depression falls within its priorities.

Consistent with findings of this study, adequate funding and resources are necessary to implement the SPADe in local antenatal clinics. This is corroborated by a study which found that additional funding enabled hospitals to increase midwifery services to support screening of perinatal mental disorders in Australia [40]. However, reduced levels of funding impedes sustainability of mental health services in low income countries [41] such as Malawi. Therefore allocating adequate financial, human and material resources will enable midwives to effectively screen for depression in antenatal clinics. This study showed that having a resource such as the SPADe’s manual enabled midwives to screen for depression in antenatal clinics. Midwives used the SPADe’s manual as a guide or reference when providing mental health care to pregnant women. There is evidence which showed that having a standardised instrument enabled screening of perinatal mental health problems in Ethiopia [41].

Despite the implementation of the SPADe being feasible, midwives reported a need for specific training; ongoing support and supervision; and additional material resources to deliver the SPADe to address the barriers for screening for depression in antenatal care settings locally. In agreement with this study, the literature indicated that limited training among health workers was found to be a barrier to screening for perinatal mental disorders in Ethiopia [41]. It is evident that training is essential for general health care workers to properly screen for mental disorders [1, 21, 33, 37, 38] in antenatal clinics. Mental health training can help midwives to acquire necessary skills [42] and improves their capacity to care for pregnant women holistically [3, 43]. In Nepal, midwives reported that mental health training prepared them to screen, treat and make appropriate referrals for women [38]. However, training alone is not adequate as health care workers require ongoing support and supervision to screen for mental disorders [38]. In this study, midwives reported that the need for ongoing support and supervision was a barrier to the successful implementation of the SPADe in the local setting. This is supported by evidence which showed that lack of clinical support and supervision were barriers for midwives screening for perinatal mental disorders [42]. Similarly, the lack of a supportive system was considered as a barrier to the successful implementation of screening for perinatal mental disorders by health care workers in Ethiopia [41]. This may imply that ongoing support and clinical supervision of midwives by mental health specialists, colleagues and managers would be crucial for the successful implementation of the SPADe. There is evidence which showed that a lack of support from colleagues or managers is one of the barriers which midwives experience when dealing with perinatal mental health issues [44]. However, organisational support may assist midwives to acquire the necessary knowledge and skills for providing perinatal mental health care [42]. Therefore it is important that barriers to the successful implementation of the SPADe should be addressed to create a suitable environment for the provision of perinatal mental health care in antenatal clinics. This can be done by adopting task-sharing approaches which have proven feasible and acceptable for midwives to integrate screening in antenatal care [32] such as the Stepped Care for Maternal Mental Health Model [45] and Thinking Healthy Psychosocial Intervention [46]. In addition, Group Antenatal Care (GANC) was considered as a framework for task sharing which was potentially feasible and acceptable for integrating mental health screening and services into maternal health care at the community level in rural Mali [32]. GANC is currently being implemented in Malawi in a clinical trial [47] and it is anticipated that the SPADe will be integrated in GANC in future as part of a scaling up process. This approach will provide an opportunity for implementing locally informed and delivered screening programmes in the country.

An intervention has to be acceptable by stakeholders before it can be adopted and implemented. Simple screening programmes enable midwives to easily implement perinatal mental health services [41]. Consistent with findings of this study midwives reported that the SPADe would be easy to use; the wording of the SPADe was clear and unambiguous; the SPADe would be easy to remember to use; the environment where they work in would not make it difficult for them to use the SPADe; and that their colleagues would support use of the SPADe. This confirms the acceptability of using SPADe among midwives in the local setting after receiving training. Consistent with this, a study of 15 midwives found that midwives considered screening for depression during pregnancy as acceptable and important in the UK [15]. This is corroborated by a systematic review (included studies = 29) which reported that clinicians consider screening of perinatal depression as acceptable [21]. Nonetheless, some midwives may consider screening for depression during pregnancy as overwhelming [14] and consequently avoid screening pregnant women. Conversely, midwives may likely engage in screening for depression if they find a screening instrument acceptable and useful in their work.

In this study, midwives reported that the SPADe would be useful to their practice and their clients would benefit from use of the SPADe. This may help in integration of screening for depression in antenatal clinics [25]. Integrating protocols such as the SPADe within existing systems and processes may facilitate their use [48] by midwives. The integration of the SPADe in antenatal clinics may promote systematic and standardised screening for antenatal depression and improve early detection and treatment of depression. It is imperative that pregnant women, who are identified by midwives as having depressive symptoms, receive appropriate treatment or referral. In Malawi, there is limited or no access to mental health services in antenatal clinics and referral pathway are not well established. Therefore it would be unethical to screen pregnant women without providing them access to treatment [38]. The current practice is that pregnant women with mild and moderate depression are put on watchful waiting without any treatment while those with severe symptoms are referred to psychiatric units which are usually far from primary care settings. Nonetheless, midwives can be trained to provide counselling which can be beneficial to women with mild and moderate symptoms of antenatal depression [39]. This would remove the need for making referrals to psychosocial counsellors [38]. Regardless, midwives still need continuous supportive supervision from mental health specialists for them to implement SPADe competently and with confidence.

### Strengths and limitations of the study

The strengths of this study are twofold. Firstly, this is the first study to document the acceptability and feasibility of screening antenatal depression by midwives using a protocol such as the SPADe in the local setting. Secondly, the study used valid and reliable instruments to collect data namely: the SAFE and OADRI. This study is limited in that it may have suffered from a training effect because data were collected immediately after the SPADe training that was conducted by the authors. However, this was minimized by using a self-reported questionnaire which was administered by research assistants. The use of a small sample size might have affected significance which was minimised by using the whole population as a sample for this study.

## Conclusion

This study proposes that it is acceptable and feasible for midwives to implement the SPADe in antenatal clinics. Midwives who will be screening for antenatal depression using the SPADe will need ongoing training, support and clinical supervision. There is also a need for an expansion of the scope of practice for midwives through task sharing in the local setting and the enhancement of service integration and collaboration between antenatal and mental health service.

## Data Availability

All data generated or analysed during this study are included in this published article.

## Abbreviations

COMREC: College of medicine research and ethics committee
GANC: Group antenatal care
GCP: Good clinical practice
OADRI: Ottawa acceptability of decision rules instrument
SAFE: Structured assessment of feasibility
SPADe: Screening protocol for antenatal depression
